# Potential antiviral effects of the marine probiotic *Paraliobacillus zengyii* on the respiratory syncytial virus

**DOI:** 10.1002/mlf2.70015

**Published:** 2025-06-18

**Authors:** Qianjin Fan, Beijie Li, Lan Chen, Mengqi Jiao, Zhijie Cao, Kun Yue, Haoyue Huangfu, Hui Sun, Xiaoxia Wang, Xuelian Luo, Jianguo Xu

**Affiliations:** ^1^ School of Medicine, Institute of Public Health, Nankai University Tianjin China; ^2^ National Key Laboratory of Intelligent Tracking and Forecasting for Infectious Diseases, Chinese Center for Disease Control and Prevention, National Institute for Communicable Disease Control and Prevention Beijing China; ^3^ Center of Reverse Microbial Etiology, School of Public Health, Shanxi Medical University Taiyuan China; ^4^ Research Unite for Unknown Microbe, Chinese Academy of Medical Sciences & Peking Union Medical College Beijing China; ^5^ Clinical & Central Laboratory of Sanya People's Hospital Sanya China

**Keywords:** antiviral activity, marine probiotics, *Paraliobacillus zengyii*, respiratory syncytial virus, type I interferon

## Abstract

Probiotics can reduce the incidence of respiratory syncytial virus (RSV) disease in premature infants; this approach is resource‐intensive and less expensive than other strategies and easier to implement than most current methods worldwide. Traditional lactic acid‐producing bacteria are the main probiotics that have been studied for RSV treatment. Marine probiotics promote the survival, immunity, and disease resistance of aquatic plants and animals. However, relatively little research has been conducted on viral infections in humans. Here, we report a slightly halophilic and extremely halotolerant marine bacterium, *Paraliobacillus zengyii*, which has antiviral activity and grows at a relatively low temperature (28°C). We found that *P. zengyii* inhibited RSV infection by regulating the interferon (IFN) response both in vitro and in vivo. *P. zengyii* significantly increased the RSV‐induced phosphorylation of TBK1 and IRF3 and the expression of antiviral factors interferon‐induced transmembrane protein 1 (IFITM1) and interferon‐induced transmembrane protein 3 (IFITM3). Furthermore, *P. zengyii* upregulated Sendai virus (SeV)‐ and poly(I:C)‐induced IFN‐β expression. These results indicate that the marine bacterium *P. zengyii* inhibits RSV infection and increases IFN‐β production in response to RSV, SeV infection, or poly(I:C) stimulation. Consequently, *P. zengyii* has potential as a broad‐spectrum anti‐RNA virus probiotic.

## Impact statement

Marine probiotics are an underexplored bioactive resource, and to date, few studies have investigated their antiviral effects. Here, we show that the marine probiotic *Paraliobacillus zengyii* inhibits respiratory syncytial virus (RSV) infection in vitro and in vivo. In addition, *P. zengyii* promotes the host innate immune response to RNA viruses and double‐stranded RNA (dsRNA) mimics. Therefore, *P. zengyii* can exert broad‐spectrum anti‐RNA virus effects, and it is a unique potential probiotic because it is slightly halophilic and extremely halotolerant. This is the only known probiotic with antiviral potential that can be grown at low temperatures, which may have advantages under certain conditions. Notably, *P. zengyii* is a natural microbial dweller of marine ecosystems isolated from animal feces.

## INTRODUCTION

Probiotics, including bacteria and other microbes, can be ingested orally or applied topically for dietary and various medicinal purposes and can protect against disease‐causing microorganisms^1^, ^2^. Researchers are currently investigating the use of probiotics to prevent diseases caused by pathogenic bacteria or viruses through mechanisms such as inhibition of pathogen adhesion to the intestinal epithelium^3^, direct inhibition of bacterial pathogens or viruses, production of short‐chain fatty acids (SCFAs), maintenance of the immune response in the intestine, and downregulation of pro‐inflammatory cytokines^4^.

The best‐known probiotic microorganisms include *Lactobacillus*, *Bifidobacterium*, *Bacillus*, *Streptococcus*, *Escherichia coli*, *Saccharomyces*, *Enterococcus*, *Propionibacterium*, and others^5^, ^6^. Some probiotics, known as marine probiotics, have been isolated from marine environments and have shown benefits for the aquaculture industry or human health^2^, ^7^, ^8^. However, few studies have investigated the antiviral effects of marine probiotics.

The genus *Paraliobacillus* comprises Gram‐positive, rod‐shaped, slightly halophilic, facultatively anaerobic, spore‐forming, and extremely halotolerant bacteria isolated from decomposing seaweed, a natural microbial dweller of the marine ecosystem^9^. In this study, we found that *Paraliobacillus zengyii*, a new member of *Paraliobacillus*, has potential antiviral effects on respiratory syncytial virus (RSV) by increasing IFN‐β production and antiviral factor expression in vitro and in vivo. Moreover, *P. zengyii* potentiates poly(I:C)‐ and Sendai virus (SeV)‐triggered IFN‐β production. Our results reveal that *P. zengyii* is a promising marine probiotic candidate with broad‐spectrum activity against RNA viruses.

## RESULTS

### *P. zengyii* inhibits RSV infection in vitro

We first evaluated the effects of *P. zengyii* on the viability of A549, Hep2, HEK‐293T, and HeLa cells. The results of the Cell Counting Kit‐8 (CCK‐8) assays revealed that the viability of the four kinds of cells was not affected by coincubation with *P. zengyii* at multiplicities of infection (MOIs) of 10, 100, or 500 CFU per cell (Figure S1A–D).

**Figure 1:**
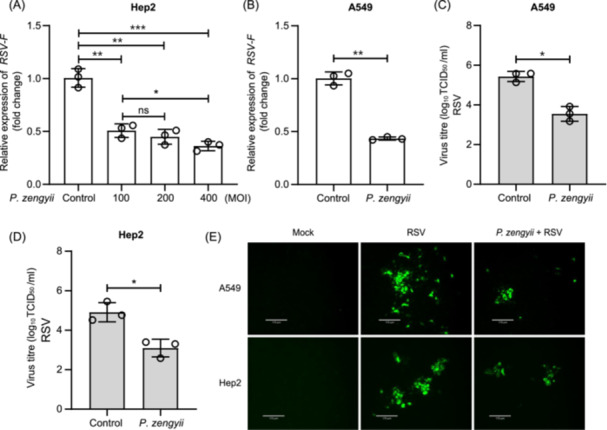
*Paraliobacillus zengyii* reduces respiratory syncytial virus (RSV) infection. (A, B) RT‐qPCR analysis of RSV‐specific gene expression in Hep2 cells (A) and A549 cells (B). (C, D) Determination of RSV titers in the supernatants of cells by a TCID_50_ assay in A549 cells (C) and Hep2 cells (D). (E) RSV fluorescence signals in A549 and Hep2 cells monitored by IFA. A549 and Hep2 cells were pretreated with or without *P. zengyii* at multiplicities of infections (MOIs) of 100, 200, or 400 CFU per cell for 24 h and then infected with RSV for 48 h. *P. zengyii* was pretreated at MOIs of 100, 200, and 400 CFU per cell in (A) and at an MOI of 100 CFU per cell in (B−E). Nuclei were visualized by DAPI staining. Images were taken using fluorescence microscopy (ECHO Revolve). Scale bars, 170 μm. GAPDH was selected as the internal reference gene for PCR quantification. IFA, immunofluorescence assay; Mock, uninfected cells. The data are shown as the means ± SD of three independent experiments. Two‐tailed unpaired Student's *t* test was used. ns, not significant; *p < 0.05; **p < 0.01; ***p < 0.001.

Next, we investigated the potential inhibitory effects of different doses of *P. zengyii* on RSV. As shown in Figure 1A, the antiviral effect of *P. zengyii* at an MOI of 400 was slightly higher than that at an MOI of 100, as demonstrated by the RT‐qPCR results, but these differences were not particularly obvious. Given the safe dosage of *P. zengyii*, we selected an MOI of 100 for subsequent assays. We subsequently identified changes in the viral load, viral titer, and GFP fluorescence signal of RSV after *P. zengyii* pretreatment of two epithelial cell lines A549 and Hep2. Compared with the control, pretreatment with *P. zengyii* reduced the viral load by as much as 50% (Figure 1A,B). The viral titer was 1.8 log_10_(TCID_50_/ml) lower than that of the control (Figure 1C,D). The fluorescence signal was significantly reduced after *P. zengyii* pretreatment (Figure 1E). In addition, we evaluated the effect of *P. zengyii* treatment duration on RSV in Hep2 cells. As shown in Figure S2A, no notable difference was found in the anti‐RSV effect of *P. zengyii* among the three pretreatment time (24, 48, and 72 h). Thus, these results indicate that *P. zengyii* can inhibit RSV infection in epithelial cells.

### *P. zengyii* increases RSV‐stimulated IFN‐β expression

**Figure 2:**
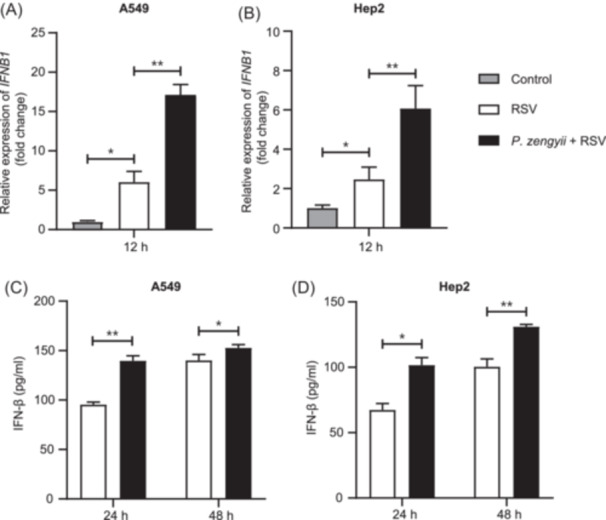
*P. zengyii* promotes the production of IFN‐β in RSV‐infected epithelial cells. (A, B) RT‐qPCR analysis of *IFNB1* mRNA in A549 cells (A) and Hep2 cells (B). Cells were pretreated with or without *P. zengyii* (MOI of 100) for 24 h and then infected with RSV (MOI of 0.1) for 12 h. (C, D) ELISA quantification of IFN‐β secretion in A549 cells (C) and Hep2 cells (D). Cells were pretreated with or without *P. zengyii* (MOI of 100) for 24 h and then infected with RSV (MOI of 0.1) for 24 h or 48 h. GAPDH was selected as the internal reference gene for PCR quantification. ELISA, enzyme‐linked immunosorbent assay. The data are shown as the means ± SD of three independent experiments. Two‐tailed unpaired Student's *t* test was used. *p < 0.05; **p < 0.01.

*P. zengyii* does not adhere to cells and is sensitive to antibiotics (100 U/ml penicillin, 100 µg/ml streptomycin, and penicillin–streptomycin) (data not shown). Therefore, the *P. zengyii* pretreatment results ruled out the direct effect of *P. zengyii* on RSV. Type I interferons (IFN‐I) play crucial roles in the antiviral response to probiotics^10^. Therefore, we evaluated the IFN‐β levels in the supernatants of the RSV‐infected A549 and Hep2 cells pretreated with *P. zengyii*. As shown in Figure 2, compared with the control, *P. zengyii* pretreatment significantly increased the mRNA expression of *IFNB1* at 12 h post‐infection (hpi) (Figure 2A,B) and the secretion of IFN‐β at 24 and 48 hpi (Figure 2C,D). These results indicate that *P. zengyii* inhibits RSV infection by promoting the production of IFN‐β in vitro.

### *P. zengyii* promotes RSV‐induced TBK1 and IRF3 phosphorylation

**Figure 3:**
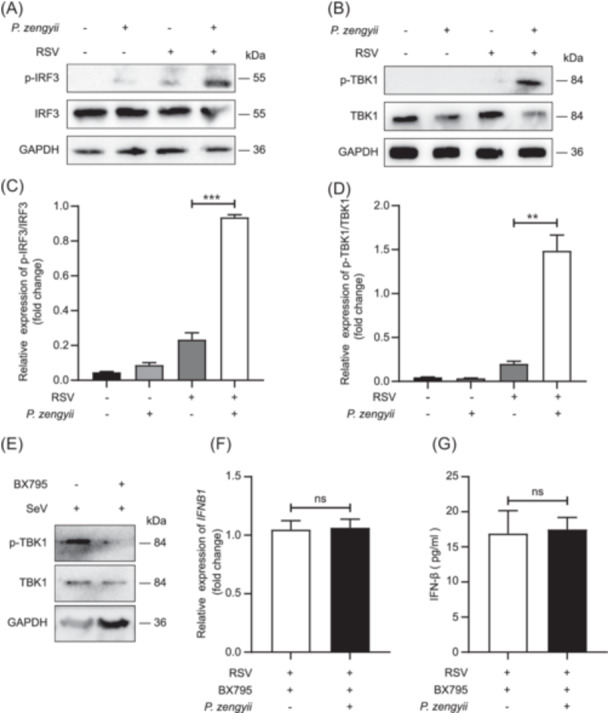
*P. zengyii* increases IFN‐β production through the TBK1–IRF3 signaling pathway. (A) Western blot analysis of IRF3 and p–IRF3 in Hep2 cells. (B) Western blot analysis of TBK1 and p–TBK1 in Hep2 cells. The cells were pretreated with or without *P. zengyii* (MOI of 100) for 24 h, followed by infection with RSV (MOI of 0.1) for 6 h. GAPDH was used as the internal reference protein. (C, D) Densitometric analysis of immunoblots showing the relative expression of p–IRF3/IRF3 (C) in (A) and p–TBK1/TBK1 (D) in (B) via ImageJ software. (E) Western blot analysis of lysates harvested from Hep2 cells pretreated with the TBK1 inhibitor BX795 (0.5 μM) and then infected with SeV (MOI of 0.1) for 6 h. (F) RT‐qPCR analysis of *IFNB1* mRNA. (G) ELISA quantification of IFN‐β. Hep2 cells were pretreated with the TBK1 inhibitor BX795 (0.5 μM) for 6 h, treated with or without *P. zengyii* (MOI of 100) for 24 h, and then infected with RSV (MOI of 0.1) for 24 h. *GAPDH* was selected as the internal reference gene for PCR quantification. The data are shown as the means ± SD of three independent experiments. Two‐tailed unpaired Student's *t* test was used. ns, not significant; **p < 0.01; ***p < 0.001.

Since the role of the TBK1–IRF3 signaling pathway‐mediated antiviral response has been established, we hypothesized that *P. zengyii* affects IFN‐β production by regulating phosphorylated TBK1 (p‐TBK1) and p–IRF3. Therefore, we investigated whether *P. zengyii* activates the TBK1 and IRF3 phosphorylation signaling pathways in RSV‐infected Hep2 cells. Hep2 cells were pretreated with *P. zengyii* for 24 h, infected with RSV for 6 h, and then harvested to prepare total cell lysates. The levels of p–TBK1 and p–IRF3 were assessed by western blot analysis. Increased expression of p–TBK1 and p–IRF3 was found in the cells pretreated with *P. zengyii* (Figure 3A–D). Moreover, TBK1 inhibitor BX795 was used to block the phosphorylation of TBK1 and IRF3. We first verified that treatment with BX795 blocks SeV‐induced TBK1 phosphorylation by performing western blot analysis (Figure 3E). Next, Hep2 cells were pretreated with BX795, treated with or without *P. zengyii*, and then infected with RSV. We found that IFN‐β expression was not affected by *P. zengyii* (Figure 3F,G). These results demonstrated that *P. zengyii* increases the production of RSV‐induced IFN‐β by regulating the TBK1/IRF3 signaling pathway.

### *P. zengyii* upregulates RSV‐induced the expression of interferon‐stimulated genes (ISGs)

**Figure 4:**
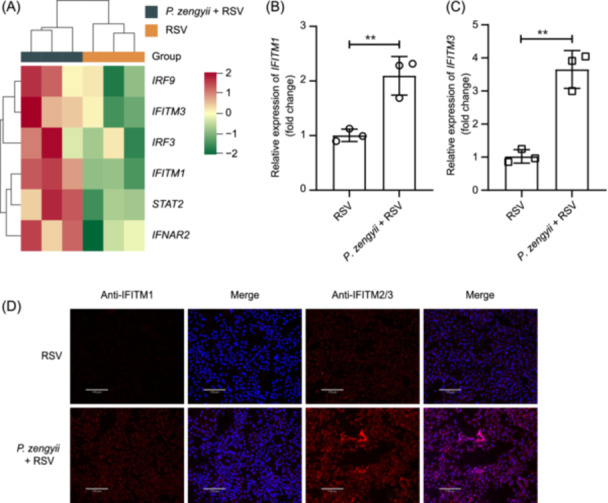
*P. zengyii* promotes the expression of ISGs in Hep2 cells. (A) Heatmap showing the expression levels of ISGs and several signaling molecules. (B, C) The mRNA levels of *IFITM1* (B) and *IFITM3* (C) determined by RT‐qPCR. (D) The expression levels of IFITM1 and IFITM3 determined by IFA. Hep2 cells were pretreated with or without *P. zengyii* (MOI of 100) for 24 h, followed by infection with RSV (MOI of 0.1) for 48 h. Nuclei were visualized by DAPI staining. Scale bars, 170 μm. IFITM1, interferon‐induced transmembrane protein 1; IFITM3, interferon‐induced transmembrane protein 3; ISGs, interferon‐stimulated genes. The data are shown as the means ± SD of three independent experiments. Two‐tailed unpaired Student's *t* test was used. **p < 0.01.

An increase in the expression of ISGs can inhibit RSV infection^25^, ^26^. To explore the potential effect of *P. zengyii* on the mRNA expression of ISGs after RSV infection, we performed transcriptomic analysis of *P. zengyii* with or without pretreatment and virus infection in Hep2 cells. As shown in Figure 4A, the transcriptomic sequencing results indicated that the mRNA levels of interferon‐induced transmembrane protein 1 (*IFITM1*) and interferon‐induced transmembrane protein 3 (*IFITM3*) were higher in the Hep2 cells treated with *P. zengyii* than in the control cells. This result was further corroborated by RT‐qPCR (Figure 4B,C). Moreover, the immunofluorescence assay (IFA) revealed that IFITM1 and IFITM3 protein expression was significantly upregulated in the Hep2 cells exposed to *P. zengyii* (Figure 4D). These results indicated that *P. zengyii* treatment upregulates IFITM expression in Hep2 cells infected with RSV.

### *P. zengyii* shows antiviral activity in mice

**Figure 5:**
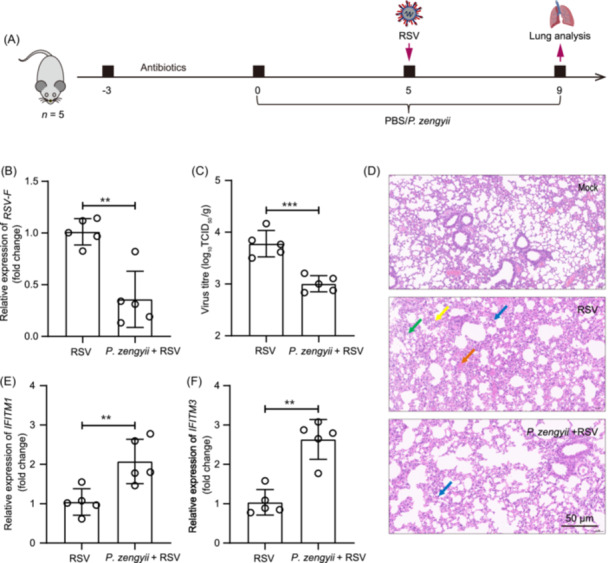
*P. zengyii* reduces RSV infection in mice. (A) Schematic representation of the animal experimental setup (*n* = 5). BALB/c mice were treated with antibiotics for 3 days and then with *P. zengyii* or PBS from 5 days before infection until 4 days after infection. (B) Viral RNA levels in the lung tissues of infected mice analyzed by RT‐qPCR. (C) Viral titers in the lungs of infected mice assessed using a TCID_50_ assay. (D) HE staining of the lungs. The alveolar wall has a small inflammatory area of cell infiltration (blue arrow), and alveolar spaces can be seen with scattered macrophages (green arrow), alveolar focal hemorrhage (yellow arrow), and minor interstitial vascular congestion (orange arrow). Scale bars, 50 μm. (E, F) The mRNA levels of *IFITM1* (E) and *IFITM3* (F) were determined by RT‐qPCR. *β‐actin* was selected as the internal reference gene for PCR quantification. Two‐tailed unpaired Student's *t* test was used. **p < 0.01; ***p < 0.001.

We next investigated whether *P. zengyii* shows anti‐RSV activity in mice (Figure 5A). Compared with that in the lungs of the RSV group, the RSV load in the lungs of the group with *P. zengyii* treatment was significantly diminished (Figure 5B). The viral titer also decreased in the *P. zengyii* treatment group (Figure 5C). Moreover, the lungs were histologically examined via hematoxylin and eosin (HE) staining. The lungs of the RSV group presented substantial alveolar wall thickening, cell infiltration, and alveolar hemorrhage (Figure 5D). However, in the *P. zengyii* + RSV group, we observed slight alleviation of pathological damage to the lungs (Figure 5D). Moreover, the expression of inflammatory cytokines, such as *IL‐6*, *IL‐1β*, and *TNF‐α*, was lower in the lungs of the *P. zengyii*‐treated RSV‐infected mice than in those of the PBS‐treated mice (Figure S3). Importantly, we examined the mRNA expression of *IFTIM1* and *IFITM3*, and found that *P. zengyii* treatment significantly increased the expression of these interferon‐induced proteins in the RSV‐infected mice (Figure 5E,F). No viral antigen was detected in the mock group (data not shown). Overall, these results suggest that *P. zengyii* confers protection against RSV infection in mice.

### *P. zengyii* promotes poly(I:C) or SeV‐induced IFN‐β production

**Figure 6:**
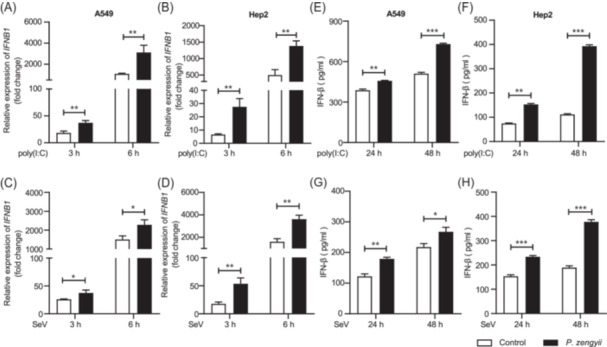
*P. zengyii* increases the production of IFN‐β in epithelial cells stimulated with poly(I:C) or infected with SeV. (A–D) RT‐qPCR analysis of *IFNB1* mRNA in A549 cells (A, C) and Hep2 cells (B, D). (E–H) ELISA quantification of IFN‐β secretion in A549 cells (E, G) and Hep2 cells (F, H). A549 and Hep2 cells were pretreated with *P. zengyii* (MOI of 100) or not for 24 h and then transfected with poly(I:C) (0.5 μg/ml) or infected with SeV (MOI of 0.1) for the indicated times. *GAPDH* was selected as the internal reference gene for PCR quantification. The data are shown as the means ± SD of three independent experiments. Two‐tailed unpaired Student's *t* test was used. *p < 0.05; **p < 0.01; ***p < 0.001.

SeV is commonly used as a potent interferon inducer^11^. Poly(I:C), a synthetic double‐stranded RNA (dsRNA), has been widely used to mimic RNA virus infection^12^. As shown in Figure 6A–D, *IFNB1* mRNA expression in A549 and Hep2 cells induced by 3 or 6 h of stimulation with poly(I:C) or infection with SeV was increased by treatment with *P. zengyii*. IFN‐β secretion induced by poly(I:C) or SeV in the supernatant was also significantly increased by *P. zengyii* treatment (Figure 6E–H). Importantly, pretreatment with *P. zengyii* reduced the viral load of SeV (Figure S2B). Therefore, these results indicated that *P. zengyii* has broad‐spectrum activity against RNA viruses.

## DISCUSSION

A randomized trial in a preterm infant population showed that compared with a placebo, probiotic supplementation decreased the incidence of viral infection^13^. Researchers have suggested that reducing the incidence of RSV disease in premature infants using a relatively cheap and accessible method, such as the administration of probiotics, is more feasible for global implementation than most current strategies, which are expensive and resource‐intensive^13^. Although further comprehensive studies are necessary to confirm the potential preventive and therapeutic effects of probiotics for RSV infection, probiotics do not appear to be associated with major adverse events. Several probiotics, such as *Bacillus* ^14^, *Lactobacillus rhamnosus* ^15^, and *L. gasseri* ^16^, have been studied for potential use in preventing RSV infection. These probiotics are present or used in fermented food product supplements. The majority of these bacteria are lactic acid‐producing bacteria isolated from humans or animals. *P. zengyii* is the first marine bacterium with anti‐RSV activity isolated from the Qinghai–Tibet Plateau.

Previous studies have shown that the replication of RSV is dose‐dependently inhibited by exogenous IFN‐I pretreatment^17^. Therefore, increasing the ability of host cells to produce IFN‐I may be an optimal strategy against viral infection. Surprisingly, *P. zengyii* inhibited viral infection by increasing IFN‐β expression in RSV‐infected cells. Notably, immunostimulatory defective viral genomes, which are often incomplete viral progeny that can stimulate IFN production, are naturally generated during RSV replication^18^, ^19^. This finding explained why RSV infection alone also induced IFN expression in the present study. Moreover, RSV infection was shown to induce the expression of inflammatory factors in the lung and causes tissue damage^20^. Our results demonstrated that *P. zengyii* alleviates the inflammation caused by RSV in mice. These results revealed that *P. zengyii* also regulates inflammation in addition to inducing IFN‐β production, which should be further studied.

TBK1–IRF3 is the main upstream signaling pathway that activates IFN. Interestingly, after pretreatment with *P. zengyii*, TBK1 and IRF3 phosphorylation significantly increased. These results revealed that *P. zengyii* mediates the production of IFN‐β associated with RSV infection via the TBK1–IRF3 signaling pathway. This mechanism has been recently confirmed by other investigators^21^, ^22^. ISGs are abundant antiviral factors induced by IFNs^23^, ^24^. More interestingly, we found that *P. zengyii* increased the expression of IFITM1 and IFITM3, which have been reported to restrict RSV infection, in RSV‐infected cells and mice^25^, ^26^. IFN‐I has broad‐spectrum antiviral effects^27^. Notably, both RSV and SeV are RNA viruses, and poly(I:C) is a dsRNA viral mimic. Therefore, *P. zengyii* may have broad‐spectrum activity against RNA viruses, and the antiviral effects on DNA viruses need further investigation. In addition, given the potential antiviral properties of probiotic‐derived metabolites or cellular components^28^, ^29^, we conducted a preliminary study to assess the antiviral activity of various extracts from *P. zengyii*, including bacterial fractions and culture supernatants. However, no significant antiviral activity was observed in any of the tested samples (data not shown). A more detailed and in‐depth analysis will be conducted in the future. Collectively, we demonstrated that the marine bacterium *P. zengyii* inhibits RSV infection by modulating the IFN response, suggesting its potential as a novel prophylactic strategy for RSV management.

## MATERIALS AND METHODS

### Bacterial strain

*P. zengyii* was isolated and stored in our laboratory^30^. The size of the genome is 3,728,451 bp, with 35.2% guanine–cytosine content. On the basis of whole‐genome sequence analysis, it was found that *P. zengyii* is phylogenetically closely related to *P. sediminis*, which was isolated from sediment from the East China Sea^31^. The bacterial strain is usually grown in BHI media supplemented with 3% (w/v) NaCl at an optimal temperature of 28°C.

### Cells, viruses, and reagents

The A549 (human adenocarcinoma alveolar basal epithelial), Hep2 (human laryngeal squamous carcinoma), HEK‐293T (human embryonic kidney), and HeLa (human cervical cancer) cell lines were obtained from the American Type Culture Collection (ATCC). A549, HEK‐293T, and HeLa cells were cultured in DMEM (Gibco). Hep2 cells were cultured in RPMI‐1640 (Gibco). The cells were cultured at 37°C under 5% CO_2_, and the medium was supplemented with 10% FBS (Vivacell) and penicillin–streptomycin (Gibco).

The RSV strain Long A (ATCC VR‐26) was propagated in Hep2 cells^32^. SeV was propagated in chicken embryos^33^. Poly(I:C) and Lipofectamine 2000 (LP2000) were obtained from Invitrogen. The TBK1 inhibitor BX795 was purchased from MCE. CCK‐8 assays were acquired from Dojindo.

### Cell viability assay

For determination of the possible cytotoxic effects of *P. zengyii*, live bacteria (MOIs of 10, 100, 500, 1000, 2000, and 5000) were added to A549, Hep2, HEK‐293T, and HeLa cell monolayers in 96‐well plates for 24 h. Then, the cell viability was determined using the CCK‐8 assay. In accordance with the manufacturer's instructions, the cell monolayers were washed after the 24 h treatment, and CCK‐8 solution was added to the cells, which were subsequently cultured for another hour. The absorbance at 450 nm was subsequently measured using an enzyme‐linked immunosorbent assay (ELISA) microplate reader. The relative percentage of cell survival was calculated in comparison with that of the control wells containing untreated cells and adjusted for background fluorescence obtained with media without cells.

### Antiviral assay

For exclusion of the direct impact of *P. zengyii* on RSV, a pretreatment experiment with *P. zengyii* was performed. A549 and Hep2 cells were seeded at a density of 3×10^5^ cells/well in 12‐well plates. The cell monolayers were then pretreated with *P. zengyii* for 24 h. Afterwards, the cells were washed three times with PBS, inoculated with RSV‐A (MOI of 0.1) or SeV (MOI of 0.1), and incubated for 2 h at 37°C for virus adsorption. The viral mixture was removed, the cells were washed with PBS, maintenance medium containing 2% FBS and penicillin–streptomycin was added, and the cells were incubated at 37°C for 48 h. Next, the cells and supernatants were collected for further analysis.

### Mouse infection

Female BALB/c mice (4 weeks old) were randomly divided into three groups: the mock group, the RSV group, and the *P. zengyii* + RSV group. The mice were treated with a mixture of antibiotics for 3 days to deplete the gut microbiota. The antibiotic cocktail included gentamicin sulfate (0.5 g/l), vancomycin hydrochloride (0.5 g/l), ampicillin (1 g/l), amphotericin B (0.1 g/l), and metronidazole (8 g/l)^34^. After antibiotic treatment, the mice in the mock, RSV, and *P. zengyii* + RSV groups were orally administered PBS (200 μl), PBS (200 μl), or *P. zengyii* (2 × 10^9^ CFU/mouse, 200 μl), respectively, daily from Day 5 before infection until euthanasia. The mice in the RSV and *P. zengyii* + RSV groups were inoculated intranasally with RSV (1 × 10^6^ TCID_50_/mouse, 40 μl), and the mice in the mock group were intranasally administered DMEM instead. At 4 days post‐infection, the mice were euthanized and their lungs were collected for subsequent analysis. HE staining was performed for histopathological analysis.

### RNA quantification

Total RNA was extracted from cultured cells or tissues with TRIzol (Invitrogen). A total of 0.5 μg of RNA was reverse‐transcribed to cDNA using a PrimeScript RT Reagent Kit (TaKaRa), and quantitative real‐time PCR was then performed with SYBR Green qPCR Master Mix (TaKaRa). The sequences of the primers used for RT‐qPCR were listed in Table S1. The results were analyzed using the Qiagen Real‐Time PCR System, and the data were normalized to the level of *GAPDH* or *β‐actin* expression in each individual sample. Relative fold changes in expression were calculated using the $2^{-\mathrm{\Delta \Delta Ct}}$ method.

### Virus quantification

The cell supernatants containing the viruses and the supernatants of the homogenized lung tissue samples were harvested after centrifugation. The RSV titer was quantified with a 50% tissue culture infectious dose (TCID_50_) assay in Hep2 cells. Briefly, the cells were seeded in a 96‐well culture plate and incubated with tenfold serial dilutions of cell supernatants or lung homogenates for 5 days. The TCID_50_ value was calculated using the Reed–Muench method.

### Immunofluorescence

A549 and Hep2 cells were pretreated with *P. zengyii* and infected with RSV (MOI of 0.1) for 48 h. Then, the cells were fixed with 4% paraformaldehyde, permeabilized with 0.2% Triton X‐100, and blocked with 5% nonfat milk. Primary antibodies were used to incubate the cells. The cells were subsequently washed with PBS and incubated with the corresponding secondary antibodies. Afterwards, the samples were washed with PBS, DAPI was added, and the cells were sealed and observed under a fluorescence microscope (ECHO Revolve). Uninfected cells were processed as mock controls. Information about all the antibodies used in our study is provided in Table S2.

### ELISA and Western blot analysis

The IFN‐β levels in the culture supernatants of A549 and Hep2 cells were measured using an ELISA kit (Proteintech). The absorbance was read by an ELISA plate reader at 450 nm. For western blot analysis, protein extracts were prepared from cells lysed with high‐strength RIPA buffer supplemented with complete protease inhibitor cocktail tablets and phosphatase inhibitor cocktail tablets PhosSTOP (Roche). Then, the protein samples were subjected to 12% SDS‐PAGE, transferred to PVDF membranes (Merck), blocked and incubated with the indicated primary antibodies at different dilutions. After washed with TBST, the membranes were incubated with an HRP‐conjugated secondary antibody. Information about all the antibodies is provided in Table S2. The proteins were visualized via enhanced chemiluminescence (ECL) reagent (TaKaRa), and densitometric analysis was performed using ImageJ software.

### RNA sequencing and analysis

Hep2 cells in different groups were pretreated with *P. zengyii* or not and then infected with RSV for 48 h. Total RNA was isolated using TRIzol reagent. Then, RNA sequencing (RNA‐seq) was performed using a BGISEQ‐500 instrument (BGI). The sequencing data were filtered with SOAPnuke (version 1.5.6) to remove reads containing sequencing adapters, low‐quality reads, and more than 5% unknown N bases; afterwards, clean reads were obtained and stored in the FASTQ format^35^. Bowtie2 (version 2.3.4.3) was used to align the clean reads to the human genome^36^. The matched data were calculated and normalized to FPKM by RSEM (version 1.3.1)^37^, and genes that were differentially expressed between samples were identified.

### Statistical analysis

Data are expressed as the means ± SD of each experiment. For determination of statistical significance, Student's *t* test was used. A *p* value of 0.05 or less indicates statistical significance. Calculations were performed using Prism software (Graphpad software 8.0).

## AUTHOR CONTRIBUTIONS

**Qianjin Fan:** Data analysis; investigation; visualization; software; formal analysis; writing of the original draft of the manuscript. **Xuelian Luo:** Investigation; resources; visualization; software; formal analysis; writing of the original draft of the manuscript. **Beijie Li:** Methodology; software; validation; visualization. **Lan Chen:** Investigation; validation; software. **Mengqi Jiao:** Investigation; validation. **Zhijie Cao:** Methodology; software. **Kun Yue:** Data analysis; visualization. **Haoyue Huangfu:** Visualization. **Hui Sun:** Visualization. **Xiaoxia Wang:** Resources. **Jianguo Xu:** Conceptualization; formal analysis; funding acquisition; methodology; resources; supervision; writing—review and editing.

## ETHICS STATEMENT

This study was carried out in strict accordance with the recommendations in the Guide for the Care and Use of Laboratory Animals. The protocol was approved by the Laboratory Animal Welfare and Ethics Committee of the National Institute for Communication Disease Control and Prevention, Chinese Center for Disease Prevention and Control (approval number 2023025).

## CONFLICT OF INTERESTS

The authors declare no conflict of interests.

## Supporting information

Supplementary Figure 1.

Supplementary Figure 2.

Supplementary Figure 3.

Supporting information.

## Data Availability

All data supporting the findings of this study are available within the article or from the corresponding author upon reasonable request.
