# Supplementary figures and images for: Potential antiviral effects of the marine probiotic Paraliobacillus zengyii on the respiratory syncytial virus
Source: mLife. 2025 Jun 18;4(3):249–58. doi: 10.1002/mlf2.70015 (PMC12207905; doi:10.1002/mlf2.70015)

(A)

**A549**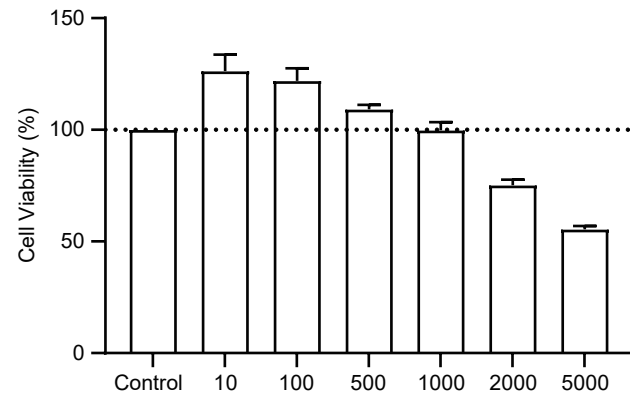

(B)

**Hep2**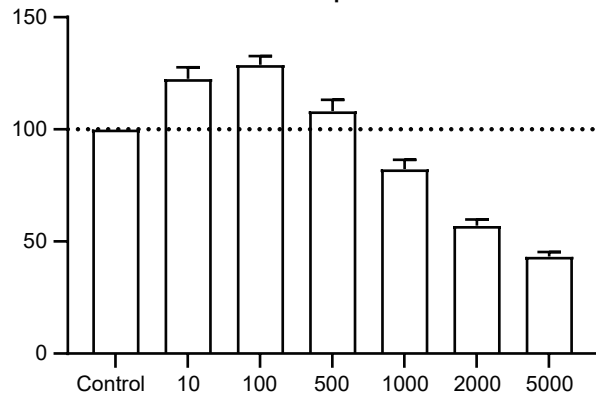

(C)

**HEK-293T**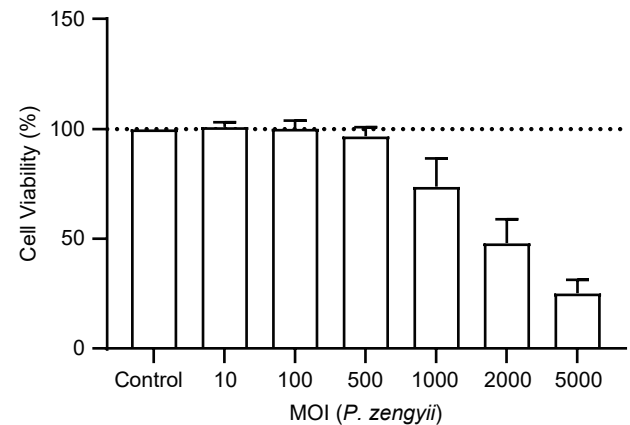

(D)

**HeLa**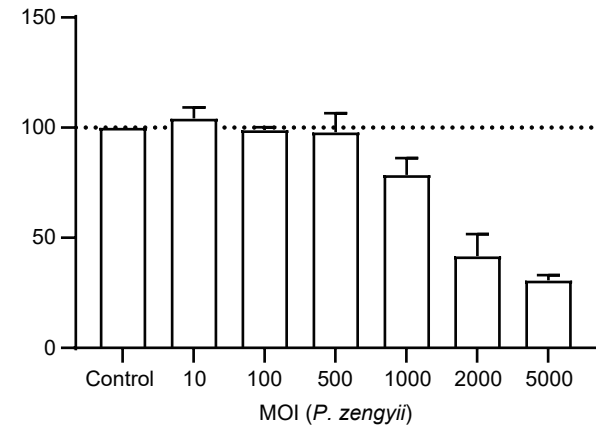

Supplement: Supplementary file 1 — Supplementary Figure 1. [file MLF2-4-249-s004.pdf]

(A)

RSV-F mRNA

Hep2

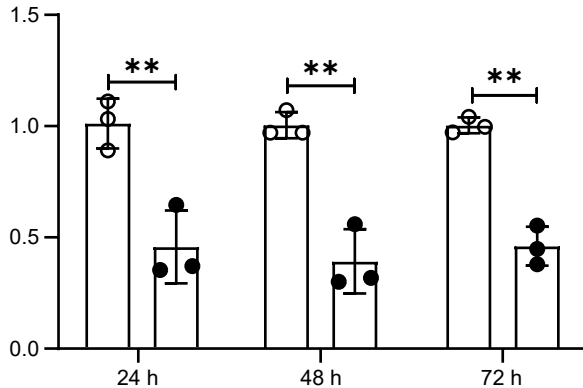

(B)

A549

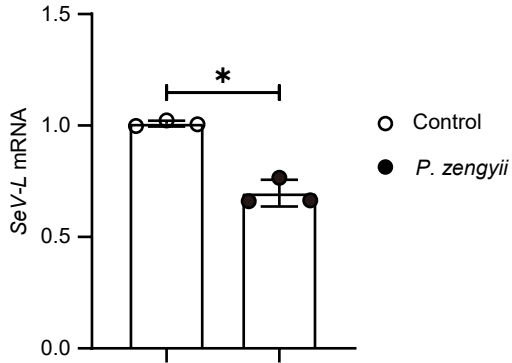

Supplement: Supplementary file 2 — Supplementary Figure 2. [file MLF2-4-249-s001.pdf]

Lung

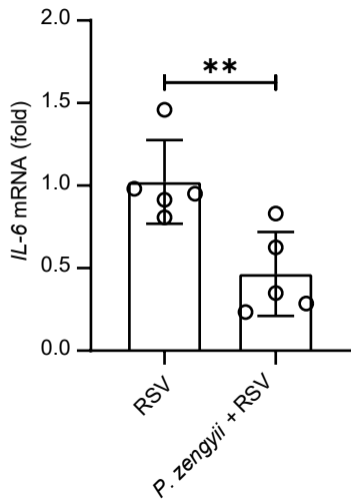

Lung

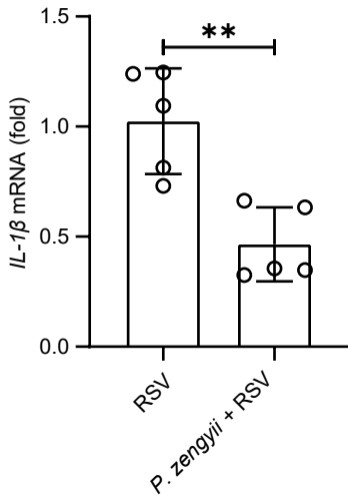

Lung

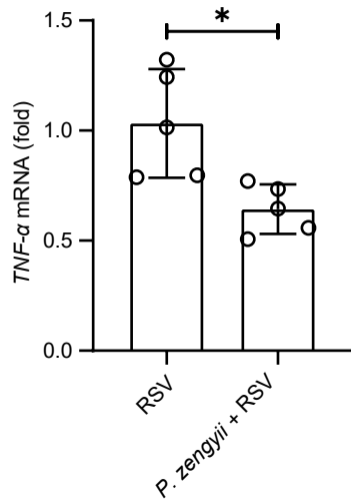

Supplement: Supplementary file 3 — Supplementary Figure 3. [file MLF2-4-249-s002.pdf]
