# Supplementary material for: Potential antiviral effects of the marine probiotic Paraliobacillus zengyii on the respiratory syncytial virus
Source: mLife. 2025 Jun 18;4(3):249–58. doi: 10.1002/mlf2.70015 (PMC12207905; doi:10.1002/mlf2.70015)
Supplement: Supplementary file 4 — Supporting information. [file MLF2-4-249-s003.docx]

**Supplementary Table 1**

Information of the primers used in this study.

| Gene name | Forward（5’‒3’) | Reverse（5’‒3’) |
| --- | --- | --- |
| *RSV‒F* | GGAACAAGTTGTTGAGGTTTATGAATATGC | TTCTGCTGTCAAGTCTAGTACACTGTAGT |
| *SeV‒L* | GCTGCCGACAAGGTGAGAGC | GCCCGCCATGCCTCTCTCTA |
| *mIL‒6* | TAGTCCTTCCTACCCCAATTTCC | TTGGTCCTTAGCCACTCCTTC |
| *mTNF‒α* | ACGTGGAACTGGCAGAAGAG | CTCCTCCACTTGGTGGTTTG |
| *mIL‒β* | TGCCACCTTTTGACAGTGATG | TGTGCTGCTGCGAGATTTGA |
| *mIFITM1* | GAAGATGGTGGGTGATACGA | GCAGCGATAGACAAGGAAAC |
| *mIFITM3* | GAGGATTCCGACTTCCGGTC | TGTTACACCTGCGTGTAGGG |
| *mβ‒actin* | CCACAGCTGAGAGGGAAATC | AAGGAAGGCTGGAAAAGAGC |
| *hIFITM1* | AGCATTCGCCTACTCCGTGAAG | CACAGAGCCGAATACCAGTAACAG |
| *hIFITM3* | ATCGTCATCCCAGTGCTGAT | ACGTGGGATACAGGTCATGG |
| *hGAPDH* | GACACCCACTCCTCCACCTTT | TTGCTGTAGCCAAATTCGTTGT |
| *hIFNB1* | GCCGCATTGACCATCTAT | TAGACATTAGCCAGGAGGTT |

m: mouse; h:human

**Supplementary Table 2**

Information about the antibodies used in this study.

| **Antibodies** | **Distributor** |
| --- | --- |
| IFITM1‒Specific Monoclonal antibody | Proteintech |
| IFITM2/3 Monoclonal antibody | Proteintech |
| Recombinant Anti‒GAPDH Antibody, Rabbit monoclonal | SinoBiological |
| Human respiratory syncytial virus (RSV) Antibody, Goat polyclonal | Sigma‒Aldrich |
| Phospho‒IRF3 (Ser396) Polyclonal antibody | Proteintech |
| IRF3 Polyclonal antibody | Proteintech |
| TBK1 Polyclonal antibody | Proteintech |
| Phospho‒TBK1 (Ser172) Recombinant antibody | Proteintech |
| Alexa Fluor® 488 conjugated anti‒goat IgG | ZSGB‒BIO |
| Alexa Fluor® 594 conjugated anti‒mouse IgG | ZSGB‒BIO |
| HRP‒conjugated anti‒rabbit secondary | ZSGB‒BIO |
| DAPI | Solarbio |

**Supplementary Figure 1.** Effect of *P. zengyii* on the viability of cells. (A) A549 cells. (B) Hep2 cells. (C) HEK‒293T cells. (D) HeLa cells. Cell viability was evaluated using CCK‒8 assays. All the values were normalized to those of the control group, which represented 100% cell viability. The cells were treated or not treated with different concentrations of *P. zengyii* (MOI of 10‒5000) at 37 °C for 24 h. The data are shown as the means ± SDs of three independent experiments.

**Supplementary Figure 2.** *P. zengyii* reduces RSV and SeV infection. (A) RT‒qPCR analysis of RSV gene expression. Hep2 cells were pretreated with or without *P. zengyii* (MOI of 100) for 24 h, 48 h or 72 h and then infected with RSV (MOI of 0.1) for 48 h. (B) RT‒qPCR analysis of SeV gene expression. A549 cells were pretreated with or without *P. zengyii* (MOI of 100) for 24 h and then infected with SeV (MOI of 0.1) for 48 h. GAPDH was selected as the internal reference gene for PCR quantification. The data are shown as the means ± SDs of three independent experiments. Two‒tailed unpaired Student’s t test was used. **P* < 0.05, ***P* < 0.01.

**Supplementary Figure 3.** *P. zengyii* downregulates proinflammatory cytokine expression in RSV‒infected mouse lungs. RT‒qPCR analysis of *IL‒6*, *IL‒1β* and *TNF‒α* gene expression in mouse lungs (n=5). β‒actin was selected as the internal reference gene for PCR quantification. IL‐6, interleukin‐6; IL‐1β, interleukin‐1β; TNF‐α, tumor necrosis factor‐α. Two‒tailed unpaired Student’s t test was used. **P* < 0.05, ***P* < 0.01.
